# Undergraduate psychiatry education: Present scenario in India

**DOI:** 10.4103/0019-5545.37311

**Published:** 2007

**Authors:** Indla Ramasubba Reddy

**Affiliations:** President, Indian Psychiatric Society; President, SAARC Psychiatric Federation; Director, VIMHANS, Vijayawada, Andhra Pradesh, India

One of the main objectives of Indian Psychiatric Society for the year 2007 is to focus on undergraduate Psychiatry education in all the medical colleges in our country. We have formed a special task force for this purpose, and we are working on this issue seriously. Let me highlight some of the reasons why Indian Psychiatric Society is very serious about Psychiatry education in the Undergraduate curriculum.

In 1835, Madras Medical College was the first medical college to be started in India. Since then, more and more medical colleges have come up. Perhaps, now it is a lucrative business to set up a medical college, and that's amply evident in the mushrooming of new private medical colleges. At present there are about 260 medical colleges, with a total of 30,000 medical seats. Four states - Andhra Pradesh, Maharashtra, Karnataka and Tamil Nadu - have 125 medical colleges, with a total of 16,000 seats. Medical Council of India is the regulatory authority, which recognizes medical colleges, lays down medical curriculum and presents standards for medical education.

The burden of mental illness on health and productivity throughout the world has long been profoundly underestimated. Data developed by the massive Global Burden of Disease study conducted by the World Health Organization, the World Bank and Harvard University reveal that mental illness, including suicide, accounts for over 15% of the burden of disease in established market economies, such as the United States.[1] This is more than the disease burden caused by all cancers.[1]

The World Health Organization's Global Burden of Disease study reported that mental disorders comprise four of the top five sources of premature death and disability in 15-44 year olds.[1] Various studies indicate that at least 20-50% of patients attending any primary-care hospital have significant psychological disorders.[2]

Many mental-health problems are typically first seen as somatic presenting complaints, and thus the general-health sector is a natural entry point for appropriately identifying and treating mental-health problems.[3] Many patients also continue to find care for psychiatric problems more acceptable when provided by their primary-care doctors.[3] There is evidence to suggest that basic mental-health services generally can be managed in primary health-care organizations with considerable cost savings and without detrimental effects on health. For this to happen, an average medical graduate should be in a position to at least identify mental disorders and refer to a specialist, if not treat the same. The willingness to approach a psychiatrist is much more if the patient is referred by a primary health-care physician. Majority of the people seek help from nonpsychiatry physicians and general practitioners. General practitioners frequently fail to detect and treat emotional distress in many of their patients. Major rift exists between Psychiatry and the rest of Medicine, which is detrimental to both the disciplines. This vacuum can be filled to a great extent by including Psychiatry education in the undergraduate curriculum. India has a huge primary health-care network. Empowering the medical officers with psychiatric knowledge during their undergraduate training can go a long way in increasing psychiatric care across the length and breadth of this huge nation.

Bhore committee in 1946 emphasized the need for training in social aspects in medical education and recommended the setting up of psychiatry departments in every general hospital.[4] The first seminar on “Undergraduate teaching in psychiatry” was held at C.I.P., Ranchi, in 1965. A national workshop on “Undergraduate medical education in mental health” was held in JIPMER in 1983. A national workshop on “Social and behavioral sciences in undergraduate training” was organized at AIIMS in 1994. However, this did not bring about any noteworthy changes in the undergraduate Psychiatry education.

The representation of Psychiatry in undergraduate curriculum is still very scant. Currently, Psychiatry is a part of Medicine and it includes about 15-20 hours of didactic lectures and two weeks of posting in Psychiatry. Psychiatry posting during internship is only optional, and that too for two weeks only. There is a nominal representation of Psychiatry in the undergraduate theory examinations and absolutely no representation in practical examinations. Many medical colleges have no departments of Psychiatry, and even many existing departments are poorly managed. There is no quality assurance in teaching and training of Psychiatry. Students are also indifferent, probably as it is not mandatory for them to learn the subject. Quite often, medical students find Psychiatry boring, and absenteeism is a major problem during Psychiatry postings. Part of the blame also rests with our own psychiatrists for probably not inspiring the students enough. Of course, it is difficult to train the undergraduates in managing all the psychiatric disorders; but at least, they should be trained in the most common psychiatric disorders which are seen in daily practice.

Sadly, there are many misconceptions about psychiatry, even among our medical colleagues. Many feel that psychiatric illness is not real and psychiatry is an inexact science. They argue that even psychiatrists cannot agree on diagnosis, and psychiatric illnesses are diagnosed by exclusion only.[2] They feel that the so-called mental diseases are only brain diseases and should be dealt by neurologists and not by psychiatrists.

We should sensitize our medical brethren, health administrators and regulatory body office bearers, policy makers about the significance of undergraduate Psychiatric education. The aim of Psychiatry training should be to empower a medical graduate to diagnose and manage common mental disorders. Indian Psychiatric Society is soon planning to organize a national-level seminar on "Undergraduate Psychiatry" involving members from Medical Council of India, Vice Chancellors of Health universities and parliamentarians, which, I am sure, will be a stepping stone in the advancement of undergraduate psychiatric education in India.
